# Palmitic acid-induced lipotoxicity promotes a novel interplay between Akt-mTOR, IRS-1, and FFAR1 signaling in pancreatic β-cells

**DOI:** 10.1186/s40659-019-0253-4

**Published:** 2019-08-19

**Authors:** Sulaiman K. Marafie, Eman M. Al-Shawaf, Jehad Abubaker, Hossein Arefanian

**Affiliations:** 1 Biochemistry & Molecular Biology Department, Dasman Diabetes Institute, P. O. Box 1180, 15462 Dasman, Kuwait; 2 Microbiology & Immunology Department, Dasman Diabetes Institute, P. O. Box 1180, 15462 Dasman, Kuwait

**Keywords:** Palmitic acid, Lipotoxicity, Insulin resistance, Type 2 diabetes, FFAR1, mTOR, Akt, IRS-1, INSR, β-cells

## Abstract

**Background:**

Free fatty acid receptor 1 (FFAR1) is G-protein coupled receptor predominantly expressed in pancreatic β-cells that is activated by a variety of free fatty acids (FFAs). Once activated, it promotes glucose-stimulated insulin secretion (GSIS). However, increased levels of FFAs lead to lipotoxicity, inducing loss of β-cell function. FFAR1 plays a key role in the development of type 2 diabetes (T2D), and previous studies have indicated the importance of developing anti-diabetic therapies against FFAR1, although its role in the regulation of β-cell function remains unclear. The present study investigated the role of FFAR1 under lipotoxic conditions using palmitic acid (PA). The rat insulinoma 1 clone 832/13 (INS-1 832/13) cell line was used as a model as it physiologically resembles native pancreatic β-cells. Key players of the insulin signaling pathway, such as mTOR, Akt, IRS-1, and the insulin receptor (INSR1β), were selected as candidates to be analyzed under lipotoxic conditions.

**Results:**

We revealed that PA-induced lipotoxicity affected GSIS in INS-1 cells and negatively modulated the activity of both IRS-1 and Akt. Reduced phosphorylation of both IRS-1 S636/639 and Akt S473 was observed, in addition to decreased expression of both INSR1β and FFAR1. Moreover, transient knockdown of FFAR1 led to a reduction in IRS-1 mRNA expression and an increase in INSR1β mRNA. Finally, PA affected localization of FFAR1 from the cytoplasm to the perinucleus.

**Conclusions:**

In conclusion, our study suggests a novel regulatory involvement of FFAR1 in crosstalk with mTOR–Akt and IRS-1 signaling in β-cells under lipotoxic conditions.

## Background

Insulin signaling is a complex multifactorial mechanism that involves various target tissues/cells, proteins, and signaling pathways. Postprandial insulin secretion occurs in a multiphasic pattern that begins with glucose uptake by target tissues (muscle and adipocytes), followed by the stimulation of lipogenesis and attenuation of hepatic glucose production [1]. Factors such as age, nutrient overload, inflammation, and adipokines affect insulin sensitivity and lead to reduced glucose uptake [2]. Type 2 diabetes (T2D) is predominantly characterized by insulin resistance, a state in which the dynamics of insulin signaling and secretion are greatly hindered [3]. Disruption of insulin secretion in β-cells has also been shown to affect insulin sensitivity [2], and insulin secretion is unable to cope with insulin resistance, triggering glucose intolerance [4].

Key players involved in insulin signaling/resistance include the insulin receptor (INSR), insulin receptor substrate 1 (IRS-1), phosphoinositide 3-kinase (PI3K), protein kinase B (Akt), and insulin. Insulin exerts its inhibitory effects by promoting ligand-induced internalization of INSR, suppressing insulin signaling and maintaining glucose homeostasis [5]. IRS-1 also plays a role in regulating insulin signaling via PI3K [6]. The PI3K/Akt pathway regulates cell cycle progression and has been implicated in β-cell mass [7]. Moreover, the mammalian target of rapamycin (mTOR) pathway (downstream of PI3K) is involved in many human diseases, including T2D. It plays important roles in cell proliferation, differentiation, and survival and is predominantly regulated by growth factors and nutrients. mTOR signaling occurs via two main complexes: mTOR complex 1 and 2 (mTORC1 and mTORC2) [8–11]. Activated mTORC1 phosphorylates its downstream target molecules P70-S6K1 and 2 (S6K1 and S6K2) and 4E binding proteins 1 and 2 (4E-BP1 and 4E-BP2) [12]. On the other hand, mTORC2 promotes phosphorylation at serine 473 of Akt, one of its main downstream targets involved in insulin signaling. The significance of mTOR signaling in T2D has been previously reported and indicates a crucial role of mTOR/S6K1 in the regulation of insulin resistance and β-cell mass and function [13].

Free fatty acids (FFAs) have also been shown to be involved in insulin resistance [14]. Palmitic acid (PA) was shown to promote mTOR signaling in rat hepatocytes [15] and also plays a key role in insulin regulation and β-cell function [16]. Increased levels of saturated FFAs affect insulin biosynthesis [17], secretion, and β-cell content [18–23] and also trigger cell stress [19, 24]. This, in turn, leads to lipotoxicity that may lead to loss of β-cell function and serves as a direct player in the pathophysiology of T2D [24–30]. FFAs bind to their main receptor, FFA receptor 1 (FFAR1), also known as GPR40, affecting insulin regulation [31, 32]. FFAR1 is a G-protein coupled receptor possessing seven transmembrane domains and is predominantly expressed in pancreatic β-cells. Activation by various medium- and long-chain (C12–C22) FFAs in β-cells triggers a signaling cascade, which leads to increased intracellular calcium levels and stimulating insulin secretion that potentiates the insulinotropic capacity of glucose, resulting in amplified glucose-stimulated insulin secretion (GSIS) [33]. However, the exact mechanism of action of FFAR1 remains unclear.

The present study investigated the role of FFAR1 in insulin signaling using the rat insulinoma 1 clone 832/13 (INS-1 832/13) cell line, a subclone of the INS-1 cells isolated by Hohmeier et al. [34]. These cells were selected as they are stably glucose-responsive and physiologically resemble native pancreatic β-cells [34, 35]. We investigated the role of FFAR1 under lipotoxic conditions using PA. We demonstrated that lipotoxicity affected GSIS, attenuated activity of both IRS-1 and Akt, and downregulated INSR1β and FFAR1. Furthermore, PA irreversibly altered the cellular localization of FFAR1. Finally, knockdown of FFAR1 affected IRS-1 and INSR1β mRNA expression. Our findings suggest potential crosstalk between Akt-mTOR, IRS-1, and FFAR1 that may help to elucidate their roles in insulin sensitivity and β-cell function involved in T2D.

## Results

### PA induces lipotoxicity in INS-1 cells

**Fig. 1:**
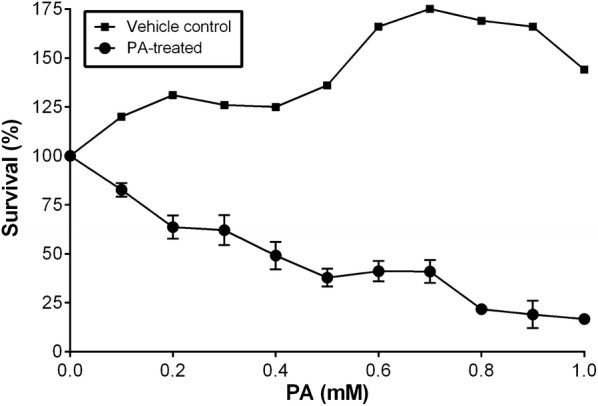
Effect of PA-induced lipotoxicity on INS-1 cells. INS-1 cells were treated with various concentrations of PA for 24 h followed by MTT cell survival assay. Triplicate cell repeats were performed for each group. The absorbance at 570 nm was measured and showed a decrease in the percentage of cell survival with increasing doses of PA compared to the vehicle control. LD_50_ was calculated as 0.4 mM PA (indicated by the dotted lines). Values represent mean ± SEM and the data are representative of at least three independent experiments

MTT cell survival assay was performed to determine the lipotoxic effects of PA on INS-1 832/13 cells. Cell viability decreased with increasing concentrations of PA, whereas no changes were observed with the vehicle controls (Fig. 1). The lethal dose 50% (LD_50_) was calculated as 0.4 mM PA as this concentration induced lipotoxicity in INS-1 cells and allowed healthy cells to continue to grow. For all experiments, 0.4 mM PA was used as a reference for PA treatments in addition to a lower and a higher dose of 0.2 mM and 0.6 mM, respectively. Different concentrations of PA were compared with untreated controls to examine its lipotoxic effects on INS-1 cells.

### PA-induced lipotoxicity affects GSIS in INS-1 cells

**Fig. 2:**
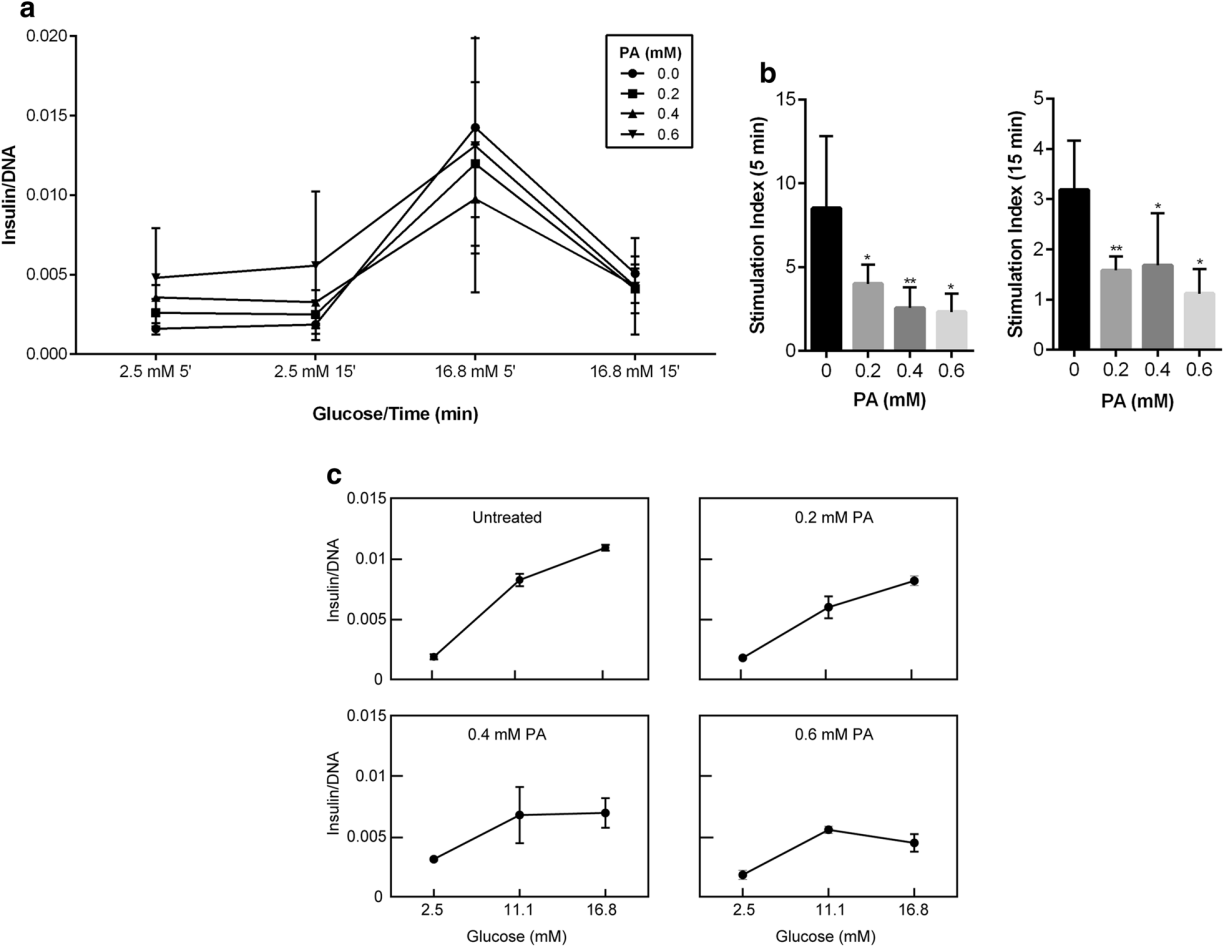
Effect of high PA concentrations on GSIS in INS-1 cells. **a** GSIS was performed using the perifusion method in which triplicate repeats of 300 clusters of INS-1 cells were pretreated with PA for 24 h and then exposed to different glucose concentrations at 5- and 15-min time points. A gradual decrease in GSIS was observed between the control and the PA-treated groups, at both low and high glucose concentrations. **b** However, the difference in both the first (5 min) and second (15 min) phase of insulin secretion was more prominent when cells were exposed to increased lipotoxic conditions (0.4 and 0.6 mM PA) indicated by their stimulation indexes. **c** A similar pattern in GSIS was observed when monolayer INS-1 cells were exposed for a longer period to different glucose concentrations using the static incubation method. Data represent mean ± SEM of normalized insulin/DNA of at least three independent experiments. *P < 0.05 and **P < 0.01

To examine the effects of PA-induced lipotoxicity on insulin secretion, GSIS was performed using two methods: perifusion and static incubation. Perifusion was performed using both 5- and 15-min time points due to biphasic insulin secretion peaking in response to glucose at these times. Data were normalized as insulin/DNA concentration where a gradual decreases in GSIS between the control (untreated) and the 0.2 mM PA group was observed both at low and high glucose concentrations. However, compared with the control, a more prominent decrease in GSIS was observed when INS-1 cells pretreated with 0.4 and 0.6 mM PA were exposed to 16.8 mM glucose levels (Fig. 2a). The decrease was further apparent by the stimulation indexes both in the first (5 min) and second (15 min) phase of insulin secretion under lipotoxic conditions where the changes were statistically significant (Fig. 2b). INS-1 cells were exposed to 50 mM KCl to trigger membrane depolarization to confirm cell function and viability under lipotoxic conditions. This was indicated by a spike in insulin secretion at both 5 and 15 min (data not shown). To confirm the reproducibility of our findings, GSIS was also performed using the static incubation method. Insulin accumulated over a 2-h period clearly showed a significant difference in GSIS compared with control cells at higher concentrations of PA upon exposure to high glucose levels (Fig. 2c). A baseline glucose concentration of 11.1 mM was introduced to demonstrate these effects at different glucose concentrations (low, basal, and high). Both methods were able to demonstrated consistent lipotoxic effects on GSIS in INS-1 cells regardless of whether cells were cultured as clusters (perifusion) or as a monolayer (static incubation).

### PA-induced lipotoxicity affects protein expression of key targets of mTOR and insulin signaling pathways

**Fig. 3:**
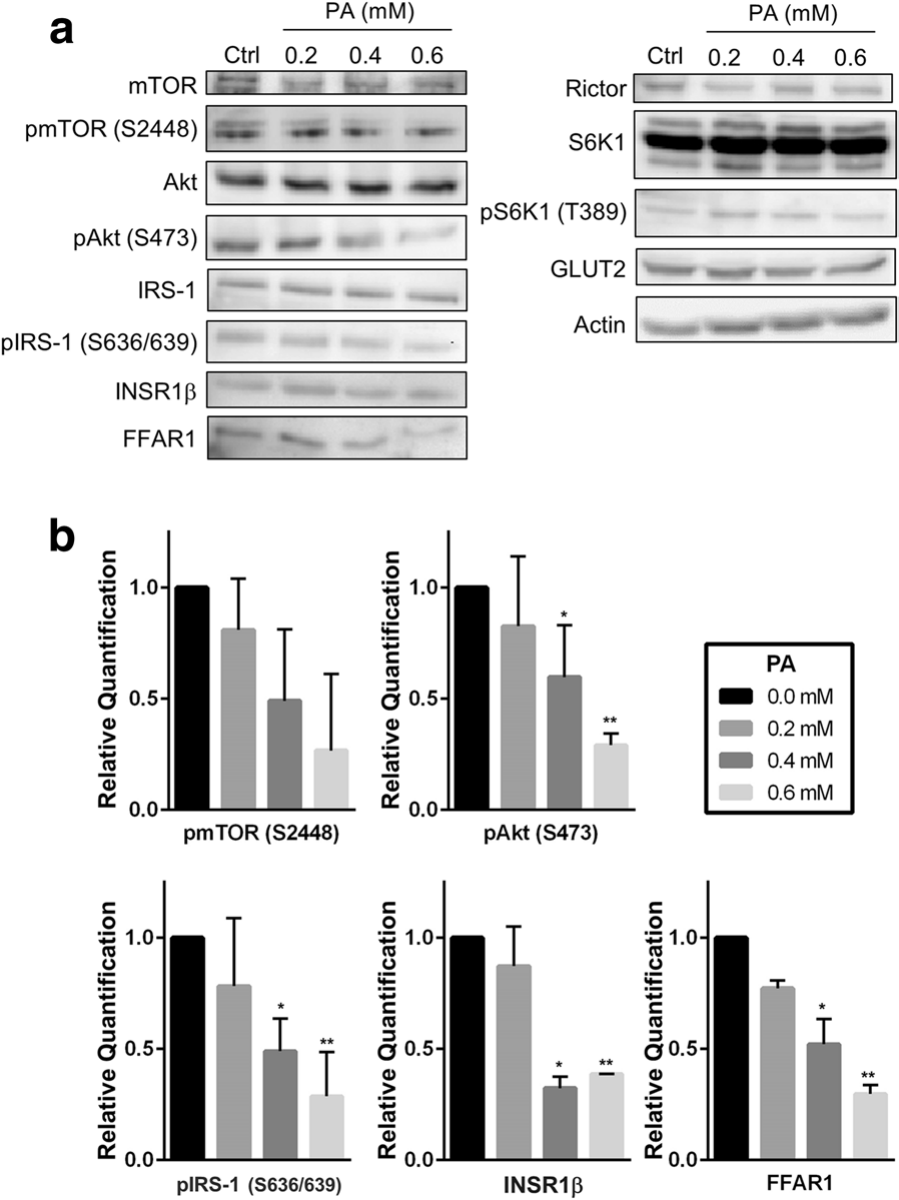
Effect of PA on protein expression in INS-1 cells. Cells were grown in a monolayer culture and treated for 24 h with different concentrations of PA. Duplicate cell repeats were performed for each group. Whole-cell lysates were separated by 8–12% SDS-PAGE and then analyzed by Western blotting. **a** The left panel illustrates both mTOR and insulin signaling pathway targets and shows a reduction in expression levels with treatment using 0.4 and 0.6 mM PA compared with the control. The right panel shows there were no significant changes in target expression with PA treatment. Actin was used as a loading control. **b** Protein quantification of targets affected by PA in (a) and their statistical significance are shown. Values represent mean ± SEM and the data are representative of at least three independent experiments. *P < 0.05 and **P < 0.01

Protein expressions of key targets of the mTOR and insulin signaling pathways were examined using Western blotting to examine the mechanisms of action PA-induced lipotoxic effects on INS-1 cells. Figure 3a shows candidate targets and their expression levels 24 h after treatment with PA. The left panel shows the negative effects of high concentrations of PA (0.4 and 0.6 mM) on expression levels of some targets compared with the untreated controls. Phosphorylation of both Akt S473 and IRS-1 S636/639 was significantly reduced by higher concentrations of PA compared with total Akt and IRS-1 levels, respectively. In addition, there was a clear reduction in FFAR1 expression under the same conditions compared with the control cells. However, phosphorylation of mTOR S2448 also decreased compared with total mTOR, but these changes were not statistically significant. Other targets of the mTOR and insulin signaling pathways, as well as the loading control, showed no significant changes (Fig. 3a, right panel). Quantification and the statistical significance of the observed changes in protein expression are shown in Fig. 3b Other targets such as Raptor, total and phospho-4E-BP1, phospho-Akt T308, and phospho-IRS-1 S307 were also examined but showed no significant changes (data not shown).

### PA-induced lipotoxicity induces FFAR1 mRNA expression

**Fig. 4:**
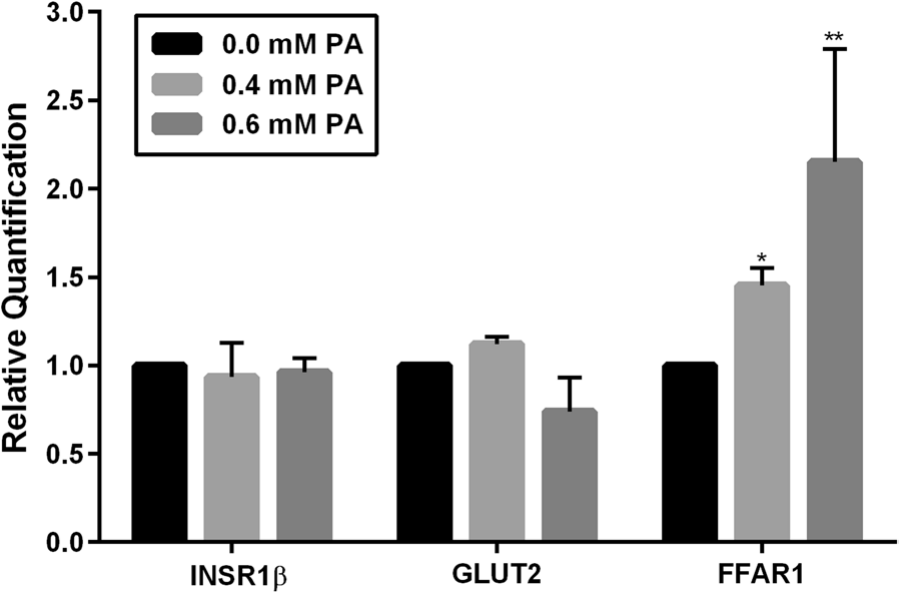
PA promotes FFAR1 mRNA expression in INS-1 cells. Cells were grown in a monolayer culture and treated with different concentrations of PA for 24 h; then, RNA was extracted and analyzed by qPCR. Duplicate cell repeats were performed for each group. No significant changes were seen in mRNA levels for both INSR1β and GLUT2 following treatment with 0.4 or 0.6 mM PA compared with the untreated controls. However, there was a clear increase in FFAR1 levels following exposure to 0.4 and 0.6 mM PA. GAPDH was used as a reference gene for normalization. Values represent mean ± SEM and the data are representative of at least three independent experiments. *P < 0.05 and **P < 0.01

The lipotoxic effects of PA at the mRNA level were analyzed using qPCR to measure mRNA expression levels of key targets. There were no significant changes in mRNA expression levels of the insulin signaling pathway targets, INSR1β and glucose transporter 2 (GLUT2), following 24-h treatment compared with the untreated controls (Fig. 4). However, there was a significant increase in FFAR1 mRNA levels following treatment with 0.4 mM PA, and a more pronounced increase (approximately 2.5-fold) in FFAR1 mRNA expression following treatment with 0.6 mM PA. PA showed differential lipotoxic effects on FFAR1 at protein (left panel, Fig. 3a) and mRNA levels (Fig. 4). These observations were reproducible and showed consistent findings. Moreover, there were no lipotoxic effects of PA on other key targets of the insulin and mTOR signaling pathways, such as IRS-1, Rictor, Raptor, and S6K1 (data not shown).

### FFAR1 knockdown affects IRS-1 and INSR1β levels in INS-1 cells

**Fig. 5:**
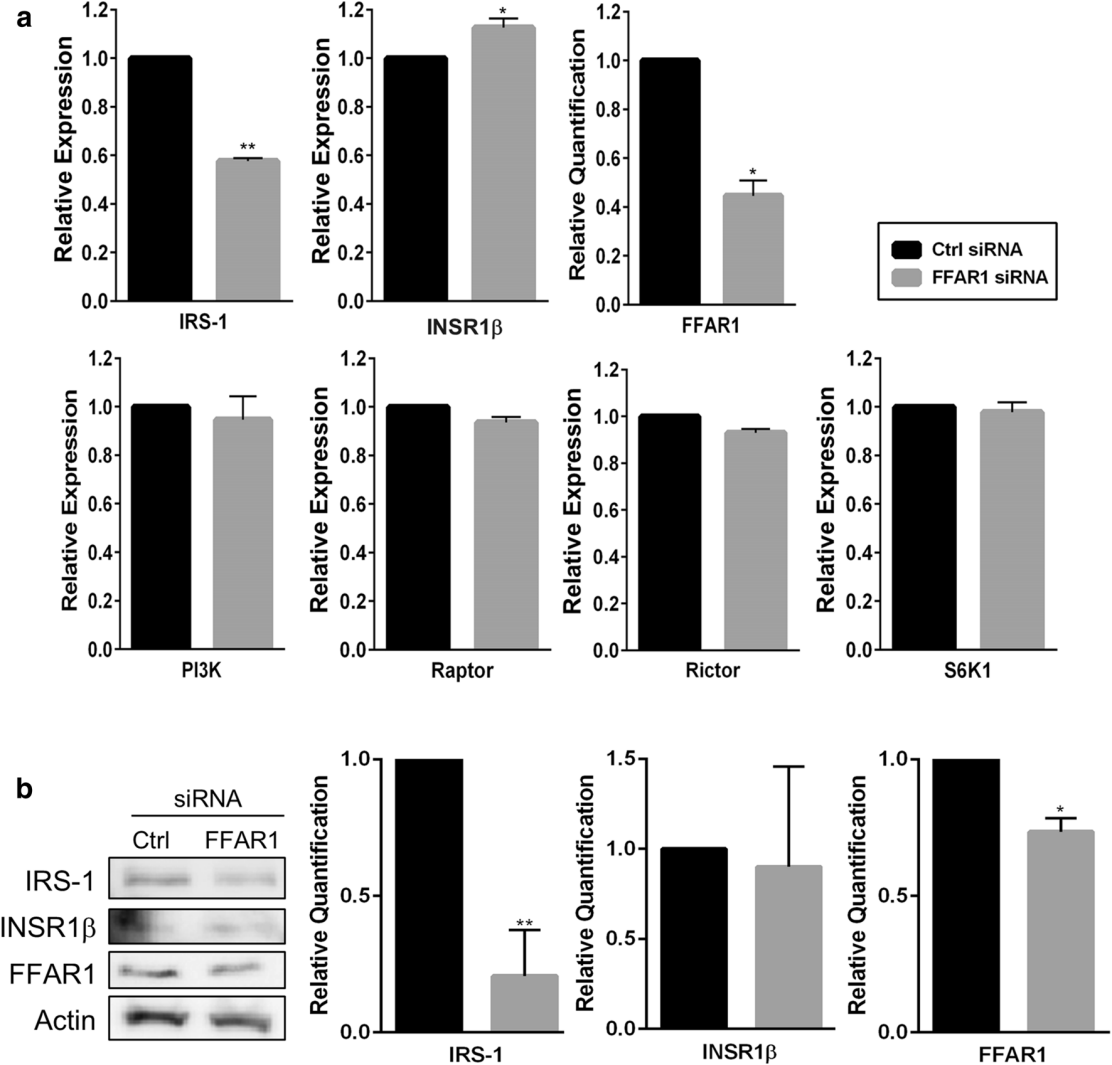
Effect of FFAR1 knockdown on mTOR and insulin signaling in INS-1 cells. Cells were transiently transfected with either 40 nM scrambled siRNA or FFAR1 siRNA for 48–72 h, harvested, and analyzed by qPCR. Duplicate cell repeats were performed for each group. Transfections yielded an efficiency of 75% (data not shown). **a** Around 60% FFAR1 knockdown was achieved on the mRNA level and led to a significant decrease in IRS-1 and an increase in INSR1β mRNA expression. Other targets of the mTOR and insulin signaling, such as PI3k, Raptor, Rictor, and S6K1, were not affected. GAPDH was used as a reference gene for normalization. **b** A representative Western blot of the protein expression and quantification is shown where approximately 30% FFAR1 knockdown was achieved that had a significant downregulatory effect on IRS-1 protein levels, but not on INSR1β. Values represent mean ± SEM and the data are representative of at least three independent experiments. *P < 0.05 and **P < 0.01

To better understand the functional role of FFAR1 in lipotoxic conditions, transient knockdown was performed using siRNA, and mRNA levels of key targets were analyzed by qPCR. Surprisingly, approximately 60% knockdown of FFAR1 mRNA levels showed a clear effect on two key targets of the insulin signaling pathway, INSR1β and IRS-1, compared with scrambled siRNA controls (Fig. 5a). IRS-1 expression levels significantly decreased in the absence of FFAR1, whereas a slight increase in INSR1β levels was observed. Other targets upstream and downstream mTOR signaling, PI3K and S6K1, respectively, as well as components of its complexes (Raptor and Rictor) showed no significant changes. On the protein level, however, approximately 30% knockdown of FFAR1 was achieved that had a significant downregulatory effect on IRS-1 protein expression as well (Fig. 5b). No significant changes were observed in INSR1β levels under the same conditions.

### Cellular translocation of FFAR1 under conditions of PA-induced lipotoxicity

**Fig. 6:**
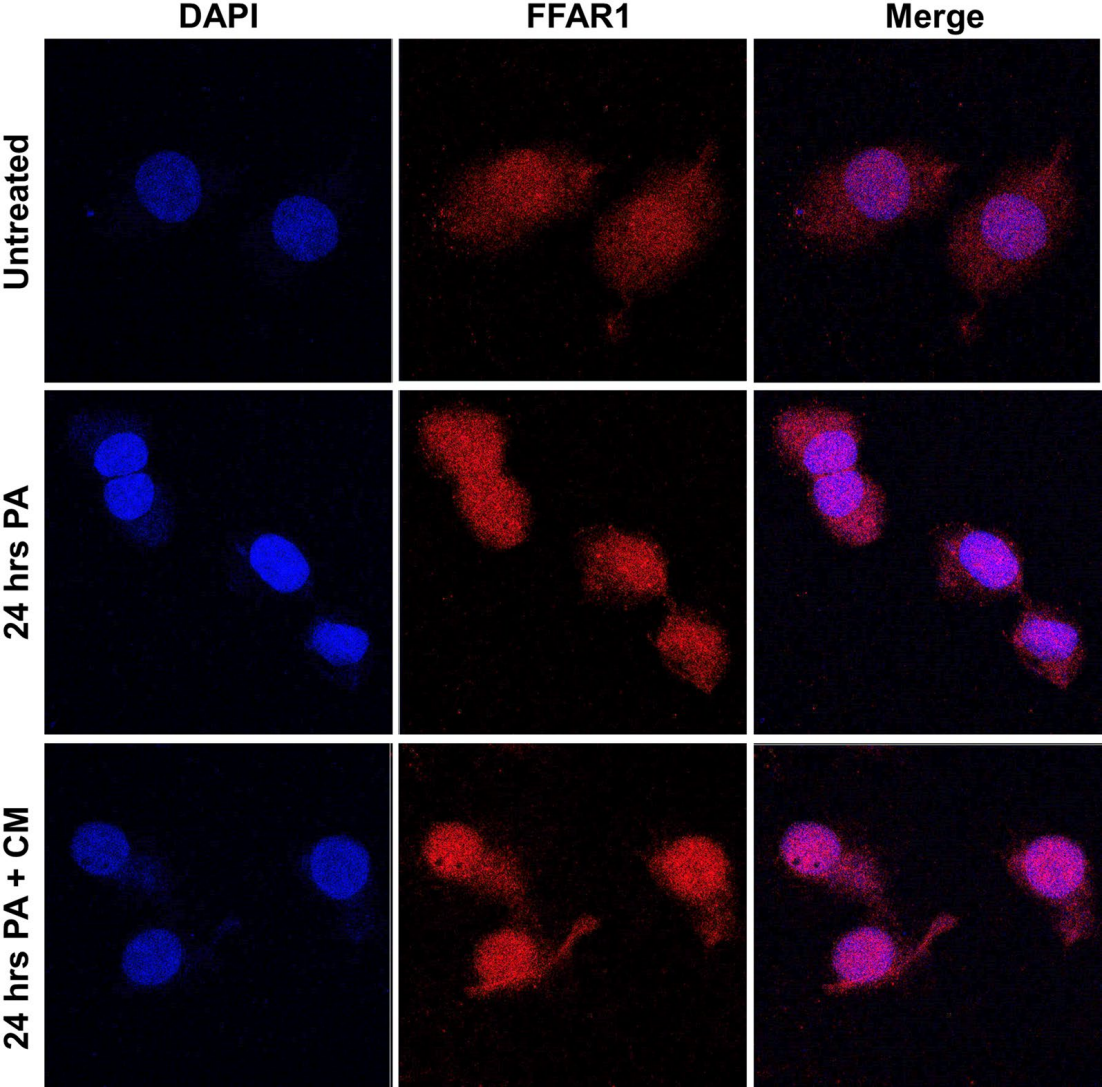
PA causes cytoplasmic to perinuclear translocation of FFAR1 in INS-1 cells. Cells were treated with 0.6 mM PA for 24 h, fixed and stained, and analyzed by confocal microscopy using a ×100 objective lens. Additionally, cells were treated with 0.6 mM PA for 24 h and then re-incubation for another 24 h with complete media. Duplicate cell repeats were performed for each group. FFAR1 conjugated with Alexa Fluor 594 (red) translocated from the cytoplasm to the perinucleus following PA treatment (middle panel), but not in the untreated cells (top panel). However, FFAR1 remained in the perinucleus despite the reintroduction of complete media (bottom panel). The mean intensity of FFAR1 for each condition was measured by ZEISS ZEN 2010 ver. 6.0 software as follows; untreated (2.9), 24 h PA (4.0), and 24 h PA + CM (4.29). Nuclei were stained using DAPI (blue). The shown data are representative of at least three independent experiments. *CM* complete media

Our findings highlighted differential expression of FFAR1 mRNA and protein levels following exposure to PA. Therefore, we investigated whether the observed changes induced by lipotoxicity influenced the cellular localization of FFAR1. Treatment of cells with 0.6 mM PA for 24 h led to the translocation of FFAR1 from the cytoplasm to the perinucleus compared with untreated controls (Fig. 6, top and middle panels, respectively). To determine whether this translocation was reversible, cells were incubated with fresh complete media for an additional 24 h following treatment with PA. However, no changes were observed, and FFAR1 remained clustered around the nucleus. The expression of FFAR1 was also measured for each group and their corresponding mean intensities are as follows; 2.9 for untreated cells, 4.0 for cells with 24 h PA, and 4.29 for cells treated to 24 h PA then reintroduced with complete media. Our results indicate that PA-induced lipotoxicity causes irreversible perinuclear localization of FFAR1.

## Discussion

The precise mechanism of FFAR1 in the regulation of β-cell functions remains elusive. The present study demonstrates a potential novel crosstalk in β-cells between FFAR1 and the Akt-mTOR pathway, a major signaling pathway involved in insulin regulation and diabetes. Knowledge of this interplay could further aid our understanding of how FFAR1 affects insulin sensitivity, insulin resistance, and overall β-cell function in T2D. FFAR1 was previously shown to be expressed in the INS-1 β-cell model [36]; however, the role of FFAR1 has not been previously investigated under lipotoxic conditions. We successfully achieved lipotoxicity in INS-1 cells and demonstrated its effect on GSIS, showing that increased levels of PA disrupted insulin secretion. It is important to optimize and control levels of PA in INS-1 since FFAs exhibit dual time-dependent effects on β-cell function and viability. It is well established that acute FFA exposure promotes GSIS, whereas chronic exposure leads to β-cell insulin resistance, dysfunction, and lipotoxicity [37, 38]. However, it remains unclear whether FFAR1 plays a role in the observed dysregulation of GSIS. To further investigate this, we selected key targets of the mTOR, Akt, and insulin signaling pathways due to their established roles in insulin secretion and β-cell function and analyzed their expression levels under lipotoxic conditions.

Several studies have associated increased mTOR activity, specifically mTORC1 activity, with an increase in β-cell size. S6K1 is a key regulator that was shown to promote β-cell size, thus affecting β-cell function, insulin content, and GSIS [39]. IRS-1 is downstream of S6K1 and is also a major player in insulin signaling that exerts its effects by regulating PI3K [40]. Furthermore, the absence of the insulin receptor in mouse β-cells caused a reduction in GSIS and promoted glucose intolerance, eventually leading to diabetes [41]. Considering the important roles of these key players in insulin signaling in maintaining β-cell function, the present study investigated whether FFAR1 also plays a role in the different pathways involved in insulin regulation. FFAR1 plays an important role in FFA-induced hyperinsulinemia. Attenuation of FFAR1 gene expression is accompanied by glucolipotoxicity in rats [42] and islets from patients with T2D [43]. This emphasizes the importance of FFAR1 signaling and its role in the development of T2D. Our results demonstrated a clear effect of PA-induced lipotoxicity on FFAR1 as well as the activity of both IRS-1 and Akt (Fig. 3). Double phosphorylation of IRS-1 at S636/639, a key sight that has been implicated in insulin resistance [44], was dramatically reduced following treatment with higher concentrations of PA. These observations were consistent and in line with a reduction of FFAR1 observed under the same conditions. Furthermore, phosphorylation of Akt at S473 was also downregulated. mTORC2 is a key regulator of Akt activity and mediates Akt phosphorylation of S473 [45]. Descorbeth et al. previously reported the effects of PA-induced lipotoxicity on Akt activity. In agreement with our findings, they also showed that PA inhibited phosphorylation of Akt at S473 in an mTORC2-dependent manner [46]. Oh et al. also demonstrated a potential link between FFAR1 and mTORC2 signaling in the context of wound healing. However, their studies were performed using FFAs other than PA and were not under lipotoxic conditions [47].

Based on our findings, we propose a possible novel link between FFAR1 and mTORC2 in pancreatic β-cells under lipotoxic conditions. One possible explanation for the downregulation of Akt at S473 is that PA-induced lipotoxicity may affect the assembly of the mTORC2 complex, affecting its kinase activity and ability to phosphorylate Akt. Yao et al. previously reported that serum withdrawal in HEK293T cells affected binding between mSin, a crucial component for mTORC2 assembly, and Akt, preventing its phosphorylation at S473 [48]. We hypothesize that similar situations might occur under lipotoxic conditions, where FFAR1 might be a key mediator in the process. However, further investigations are required to confirm our hypothesis. Both in vitro and in vivo binding experiments would be key to confirm the possible effect FFAR1 might have on the assembly of mTORC2, and consequently, affecting the activity of Akt.

Previous studies have demonstrated interplay between both mTOR complexes, where S6K1 is a key regulator of mTORC2 activity [49]. However, none of the mTORC1 signaling targets in our study showed any changes in either expression levels or activity under lipotoxic conditions. In addition, our data did not reveal any changes in the expression of the two key components of mTORC1 and mTORC2, Raptor and Rictor, respectively, or any of the upstream effectors of mTOR and Akt [PI3K and phosphoinositide-dependent kinase 1 (PDK1)]. Mordier et al. reported that PA promotes mTORC1 signaling in rat hepatocytes, and increased phosphorylation of both S6K1 and IRS-1 was observed in the presence of PA [15]. Their findings conflict with our data, possibly because different cell types show different expression patterns under PA-induced lipotoxicity. Therefore, a thorough examination comparing different model systems is crucial.

To further investigate the functional relevance of the observed changes in FFAR1 expression, we examined the effects of lipotoxicity on mRNA levels of our candidate genes under the same conditions. However, contrary to protein expression levels, FFAR1 mRNA expression was dramatically increased at higher concentrations of PA (Fig. 4), which may be attributed to posttranslational modifications (PTMs) affecting FFAR1. The notion that PTMs may affect FFAR1 expression under lipotoxic conditions prompted us to investigate whether the observed changes could affect the cellular localization of FFAR1. Indeed, PA promoted a shift of FFAR1 localization from the cytoplasm to the perinucleus (Fig. 6). It has been implicated that proteins may undergo changes in cellular localization due to PTMs during protein trafficking and/or degradation [50, 51]. Specifically, PA has been shown to induce insulin resistance and promote ubiquitination of key insulin signaling molecules such as IRS-1 and Akt [52]. Moreover, studies have demonstrated trafficking of FFAR1 to perinuclear structures in the presence of linoleic acid. FFAR1 was shown to colocalize with early endosomal markers involved in protein shuttling, namely, Rab 4 and 5, in an agonist-dependent manner [51]. We propose that FFAR1 may also undergo a similar internalization and shuttling in response to PA. It is also possible that lipotoxicity targets FFAR1 for lysosomal degradation by recruiting it to structures such as the Golgi apparatus, leading to a perinuclear pattern. This could possibly explain the reason behind the different trend in FFAR1 expression in both our immunofluorescent microscopy and Western blotting data. FFAR1 appears to have a higher protein content in the presence of PA when compared to the control under the microscope (Fig. 6), whereas the opposite is seen in the Western blots (Fig. 3). This could be attributed to the sensitivity of immunofluorescence microscopy in detecting three-dimensional epitopes. In either case, it appears that PA-induced lipotoxicity affects the cell surface as well as the internal (perinuclear) content of FFAR1. However, further investigation is necessary to confirm whether PTMs affect FFAR1's cellular distribution under PA-induced lipotoxicity.

Another surprising finding of our study was the effect of FFAR1 knockdown on key players of the insulin signaling pathway. IRS-1 mRNA and protein levels showed a distinct decrease in the partial absence of FFAR1 (Fig. 5a, b) and a slight increase in INSR1β mRNA expression (Fig. 5a). On the other hand, other targets such as PI3K, PDK1, and components of the mTOR complex showed no changes in the attenuation of FFAR1. Taken together, the protein and mRNA findings for IRS-1 may indicate potential interplay between insulin signaling and FFAR1, where IRS-1 and INSR1β are key players. To our knowledge, no previous study has reported crosstalk between these pathways, emphasizing the need to further elucidate the regulation of FFAR1 and its role in lipotoxicity. It is also important to determine whether FFAR1 regulates or is regulated by IRS-1. It is unclear whether these effects are directly or indirectly affected by mTOR signaling since the activity of key players within the pathway showed no significant changes in either protein or mRNA levels. Despite a reduction of FFAR1 protein expression and Akt activity under lipotoxic conditions, it remains unclear whether Akt acts as a regulator or is mediated by these effects. There has been increased interest in FFAR1 for the development of anti-diabetic targeted therapies for the treatment of T2D since studies have demonstrated its role in insulin resistance [53]. TAK-875 is one of the first FFAR1 agonists used in human trials and has demonstrated potent effects in improving glucose tolerance and enhancing glucose-dependent insulin secretion in vitro and in vivo [54]. However, a better understanding on the mode of action of FFAR1 and its potential partners is required to strengthen and reiterate its importance for the development of further T2D-targeted therapies.

## Conclusions

**Fig. 7:**
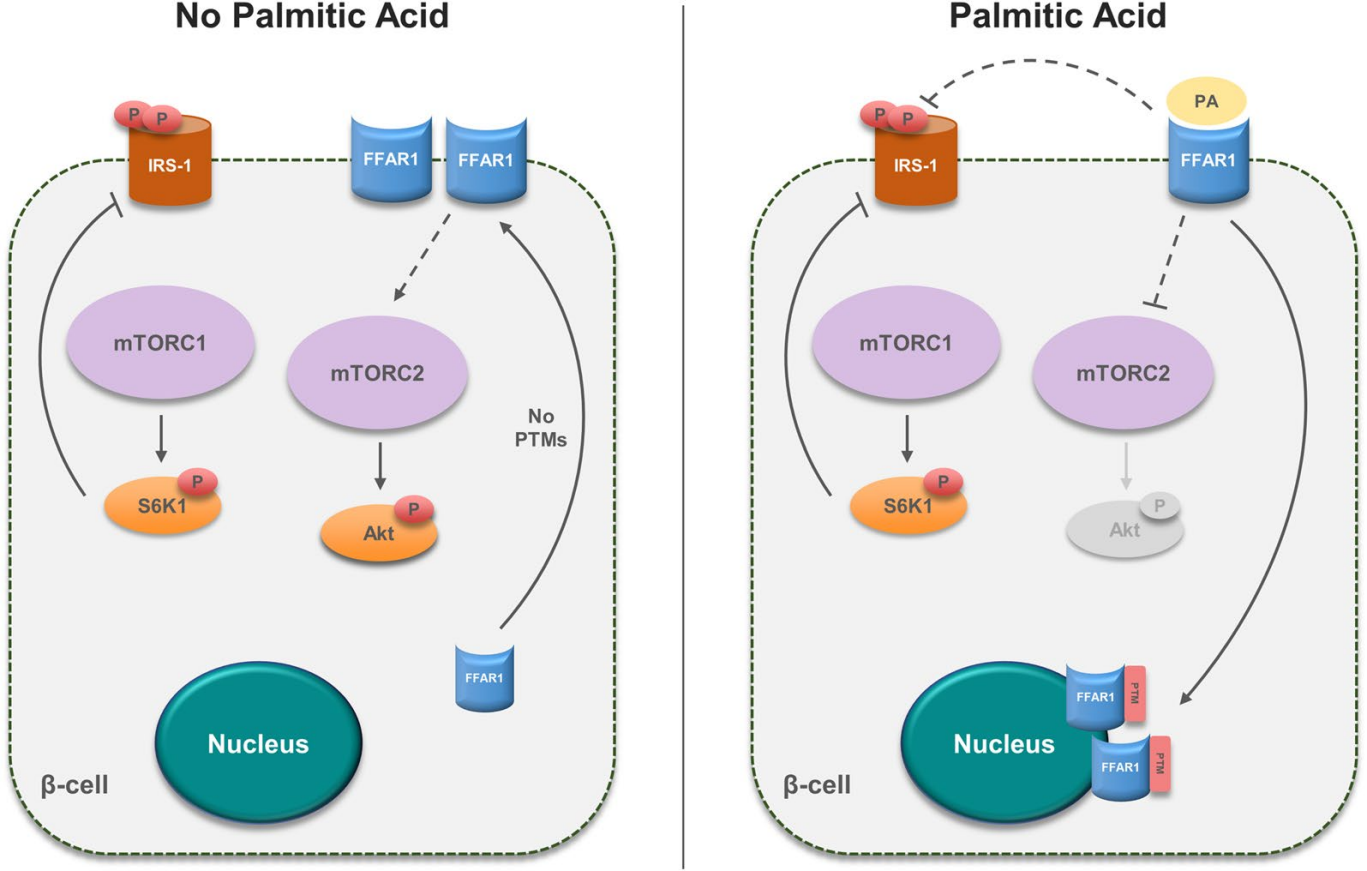
Proposed model of the crosstalk between mTORC2, IRS-1, and FFAR1 signaing in β-cell under lipotoxic conditions. In the absence of PA, FFAR1 is predominantly located at the cell surface and/or cytoplasm due to the absence of PTMs allowing it to exert its effect indirectly (dashed arrow) on mTORC2, and consequently, phosphorylating and activating Akt at S473 (left panel). On the other hand, PA-induced lipotoxicity mobilizes FFAR1 to perinuclear structures due to possible PTMs attenuating the activity of mTORC2 (dashed line). This also has an indirect inhibitory effect (dashed line) on the double phosphorylation of IRS-1 at S636/639 (right panel). *P* phosphorylation, *PTM* posttranslational modification

In conclusion, the present study demonstrates a possible new regulatory role for FFAR1 involving crosstalk between Akt-mTOR and IRS-1 signaling in β-cells under lipotoxic conditions (Fig. 7). Our results also revealed a shift in the cellular localization of FFAR1 under lipotoxic conditions, possibly mediated by PTMs. However, the suggested role of PTMs is speculative at the time of the study and further investigation is required to confirm our hypothesis. It is important to understand the exact mechanism of action of FFAR1 and the physiological relevance of our observations to better understand how FFAR1 promotes insulin resistance and the development of T2D.

## Methods

### Materials

PA was purchased from Sigma (USA). Antibodies against Rictor, Akt, phospho-Akt S473, IRS-1, phospho-IRS-1 S636/639, and INSR1β were purchased from Cell Signaling Technology (USA). Antibodies against mTOR, phospho-mTOR S2448, S6K1, phospho-S6K1 T389, and GLUT2 were purchased from Santa Cruz Biotechnology (USA). Anti-FFAR1 was purchased from Novus Biologicals (USA). Secondary antibodies used were HRP-linked (GE Healthcare, USA) and Alexa Fluor 594 (Invitrogen, USA). The non-targeting scrambled control siRNA was purchased from Dharmacon (USA) whereas all qPCR primers and the FFAR1 siRNAs were purchased from QIAGEN (Netherlands). Insulin was quantified using Insulin ELISA Kits (Chrystal Chem. INC., USA), and DNA was quantified using Quant-iT PicoGreen dsDNA Assay Kit (Invitrogen, USA).

### Cell culture and treatments

The INS-1 832/13 rat insulinoma cell line was obtained from Dr. Christopher Newgard (Duke University, Durham, USA) [34]. Cells were grown in a monolayer culture (passages 45–80); cultured in 5% CO_2_, 95% air, and 37 °C atmosphere; and maintained in regular RPMI 1640 medium containing 11.1 mM glucose supplemented with 10% fetal bovine serum, 2 mM l-glutamine, 10 mM HEPES, 1 mM sodium pyruvate, 50 μg/mL penicillin, 100 μg/mL streptomycin, and 50 μM β-mercaptoethanol (all purchased from Invitrogen, USA). PA conjugated with fatty acid-free bovine serum albumin (BSA) was prepared freshly in ethanol and used to treat cells at different concentrations for 24 h. An empty vehicle control of ethanol and fatty acid-free BSA without PA was used as a negative control (untreated).

### Cell survival

Cell survival was determined using MTT assay (Trevigen, USA) according to the manufacturer’s instructions. Plates were analyzed using Synergy H4 Hybrid Microplate Reader (BioTek, USA), and data analysis was performed using Gen5 software.

### GSIS

GSIS was performed using [1] perifusion system (Biorep Technologies, USA) and [2] static incubation method using cells pretreated with PA as described above. For perifusion, cells were grown in clustered cultures and maintained in Krebs–Ringer HEPES buffer (basal media) containing 135 mM NaCl, 3.6 mM KCl, 5 mM NaHCO_3_, 0.5 mM MgCl_2_, 1.5 mM CaCl_2_, 10 mM HEPES, and 0.1% BSA. Cells were then stimulated with different concentrations of glucose and KCl, and the flow-through was collected at 1-min intervals for 20 min. The stimulation indexes were calculated by dividing the levels of normalized insulin concentrations of the 16.8 mM glucose treatments over the 2.8 mM glucose treatments at each corresponding time point. For the static incubation method, cells were grown in monolayer cultures and treated with both glucose and KCl for 2 h. For both methods, insulin secretion was measured using ELISA and normalized to the corresponding DNA content. Extracted DNA (using QIAGEN DNA Kits, USA) was quantified using PicoGreen reagent (Molecular Probes, USA). ELISA and PicoGreen plates were measured using the Synergy H4 Hybrid Microplate Reader (BioTek, USA) and analyzed using Gen5 software.

### Western blotting

Cells were lysed with ice-cold Tris lysis buffer containing 20 mM Tris pH 7.4, 137 mM NaCl, 25 mM sodium β-glycerophosphate, 2 mM sodium pyrophosphate, 2 mM EDTA, 10% glycerol, 1% Triton X-100, 1 mM sodium orthovanadate, 1 mM PMSF, 5 μg/mL leupeptin, and 5 μg/mL aprotinin (all purchased from Sigma, USA). Whole-cell lysates were collected and quantified using the Pierce BCA Protein Assay Kit (Thermo Scientific, USA) and mixed with 4× Laemmli Sample buffer (Bio-Rad, USA). Equal amounts of protein were loaded and resolved on 8–14% SDS polyacrylamide gels followed by transfer onto PVDF membranes (Millipore, USA). The membranes were blocked with Tris-buffered saline (TBS) in 5% non-fat milk containing 1% Tween 20 (Sigma, USA) for 1 h at room temperature and then incubated with their corresponding primary antibodies overnight at 4 °C. Membranes were washed with TBS containing 0.1% Tween 20 and then incubated with their corresponding HRP-linked secondary antibodies for 1 h at room temperature. After washing, proteins were visualized using Amersham ECL Prime Western Blotting Detection Reagent (GE Healthcare, USA) and images were captured using Bio-Rad’s VersaDoc Imaging System 5000 (USA).

### Real-time qPCR

Total RNA was isolated from cells using RNA extraction kits (QIAGEN, Netherlands) according to the manufacturer’s instructions. RNA concentrations were determined using an Epoch Microplate Spectrophotometer (BioTek, USA), and qPCR samples were prepared using QuantiTect SYBR Green RT-PCR Kit (QIAGEN, Netherlands). The qPCR reactions were performed and analyzed using Applied Biosystems 7500 Fast Real-Time PCR System (Thermo Scientific, USA). Relative expression was measured using the ΔΔCT method [55].

### Gene knockdown in vitro experiments

Cells were plated at a density of 50–60% and grown in monolayer cultures and transfected with 40 nM of scrambled or FFAR1-validated siRNAs for 48–72 h using Lipofectamine 2000 (Invitrogen, USA) according to the manufacturer’s instructions. The FFAR1 siRNAs target the coding region between base pair 100–200 without any off-target effects. BLOCK-iT Fluorescent Oligo (QIAGEN, Netherlands) was used to measure transfection efficiency. Cells were then treated with PA for 24 h and harvested for qPCR analysis.

### Immunofluorescence microscopy

Cells were split on coverslips till sub-confluency and treated with PA for 24 h. In a separate experiment, cells were treated with PA for 24 h and reintroduced to complete fresh media for a further 24 h. Cells were then fixed with 4% paraformaldehyde for 15 min at room temperature and washed with PBS. Cells were incubated in blocking buffer composed of PBS containing 1% BSA and 0.1% Tween 20 (both from Sigma, USA) for 40 min at room temperature. Cells were then incubated with primary antibodies for 1 h at room temperature followed by washing with PBS and then incubated with their corresponding Alexa Fluor-conjugated secondary antibodies for 1 h at room temperature. After washing with PBS, coverslips were mounted onto glass slides using Prolong Gold Antifade Mountant with DAPI (Invitrogen, USA). Slides were examined using a ZEISS LSM 710 confocal microscope (Germany).

### Statistical analysis

For cell survival assays, nonlinear regression was used as a parameter to calculate LD_50_. All remaining results are expressed as mean ± SEM of at least three independent repeats. Data were analyzed using GraphPad Prism version 6.05 (CA, USA). Group means were compared using unpaired Student t-test. The differences with *P* < 0.05 were considered statistically significant.

## Data Availability

All data generated or analyzed during this study are included in this published article.

## Abbreviations

4 EB-P1/2: 4E binding protein 1/2
Akt: protein kinase B
FFA: free fatty acid
FFAR1: free fatty acid receptor 1
GLUT2: glucose transporter 2
GSIS: glucose-stimulated insulin secretion
INS-1 832/13: rat insulinoma cell line 1 subclone 832/13
INSR: insulin receptor
IRS-1: insulin receptor substrate 1
LD_50_: lethal dose 50%
mTOR: mammalian target of rapamycin
mTORC1/2: mammalian target of rapamycin complex 1/2
PA: palmitic acid
PDK1: phosphoinositide-dependent kinase 1
PI3K: phosphoinositide 3-kinase
PTM: posttranslational modification
S6K1/2: P70-S6 kinase 1/2
T2D: type 2 diabetes
